# Effects of high-intensity interval training on glycemic control and cardiometabolic risk factors in adults with prediabetes: a systematic review and meta-analysis

**DOI:** 10.3389/fendo.2026.1837386

**Published:** 2026-05-14

**Authors:** Jun Li, Jin Yuan, Feng Wang, Quanwen Zeng, Yong Zhang, Haifeng Ma

**Affiliations:** 1 School of Athletic Performance, Shanghai University of Sport, Shanghai, China; 2 School of Physical Education, Anhui Polytechnic University, Wuhu, China; 3 School of Physical Education, Xiangnan University, Chenzhou, Hunan, China

**Keywords:** 2-hour plasma glucose, body mass index, cardiometabolic risk factors, cardiorespiratory fitness, fasting blood glucose, glycated hemoglobin, high-intensity interval training, meta-analysis

## Abstract

**Objective:**

To systematically review and meta-analyze intervention studies evaluating the effects of high-intensity interval training (HIIT) in adults with prediabetes, with a focus on glycemic control and cardiometabolic risk-related outcomes.

**Methods:**

PubMed, Embase, Web of Science, EBSCO, and the Cochrane Library were searched from inception to January 2026. Eligible studies included adults with prediabetes who participated in structured HIIT, with either a non-exercise control or an exercise comparator. Primary outcomes were glycemic measures, and secondary outcomes were cardiometabolic risk-related indicators. Change-from-baseline values were used preferentially for pooling effect sizes. Risk of bias was assessed using the Cochrane Risk of Bias 2 tool, heterogeneity using Cochran’s Q and I², and certainty of evidence using GRADE. This review was registered in PROSPERO (CRD42024605154).

**Results:**

Thirteen intervention studies were included. Because of the limited number of eligible studies, no quantitative synthesis was performed for comparisons with non-exercise controls or across different HIIT protocols. Compared with HIIT, moderate-intensity continuous training (MICT) showed a small but statistically significant advantage for fasting blood glucose (FBG) (MD, 0.12 mmol/L, 95% CI 0.02 to 0.22; very low certainty). In contrast, HIIT showed significant advantages for 2-hour plasma glucose (2hPG) (MD, −0.16 mmol/L, 95% CI −0.28 to −0.04; low certainty) and cardiorespiratory fitness (SMD, 1.16, 95% CI 0.44 to 1.87; moderate certainty). No significant between-group difference was found for body mass index. Evidence for HbA1c, blood pressure, and lipid-related outcomes was limited.

**Conclusions:**

In adults with prediabetes, the effects of HIIT may be outcome-specific. HIIT may be more favorable for post-challenge glycemic control and cardiorespiratory fitness, whereas MICT may offer a modest advantage for fasting glucose. However, the certainty of evidence for glycemic outcomes was generally low to very low, and many included studies were short-term, suggesting that some observed effects may reflect short-term physiological responses rather than sustained clinical adaptations. Further high-quality trials with longer intervention periods are needed.

## 1 Introduction

Prediabetes is a high-risk metabolic state intermediate between normoglycaemia and type 2 diabetes (T2D), and is associated not only with a substantially increased future risk of T2D, but also with higher risks of all-cause mortality and cardiovascular disease (CVD) (1–4). Previous studies have shown that prediabetes is not merely a state of “mild hyperglycaemia”, but is often accompanied by multiple cardiometabolic abnormalities, including insulin resistance, impaired β-cell function, overweight or obesity, dyslipidaemia, elevated blood pressure, and sedentary behaviour. Therefore, the clinical management of prediabetes should not be limited to delaying diabetes onset, but should also address the early identification and comprehensive management of cardiovascular risk (1–4).

Lifestyle intervention is the first-line strategy for the management of prediabetes, and exercise is widely regarded as one of its most scalable core components (4, 5). Observational evidence suggests that higher levels of physical activity are associated with a lower risk of incident T2D and more favourable metabolic outcomes (6). In addition, systematic reviews and meta-analyses have shown that intensive long-term lifestyle intervention can significantly reduce the risk of progression from prediabetes to T2D (5). Position statements on exercise for diabetes and prediabetes have also emphasized that regular physical activity is an important pathway for improving glycaemic regulation and overall cardiometabolic health (7). Accordingly, identifying the effects of different exercise modalities on glycaemic control and related cardiometabolic outcomes during this critical intervention window is of clear clinical and practical importance.

Among the various exercise strategies, high-intensity interval training (HIIT), which alternates brief bouts of vigorous exercise with recovery periods, has attracted growing attention because of its high time efficiency and its potential to partly overcome the common time barriers and adherence limitations associated with traditional continuous aerobic exercise in real-world settings. Previous studies have shown that HIIT can improve glycaemic regulation, cardiorespiratory fitness, and some cardiometabolic outcomes. Compared with moderate-intensity continuous training (MICT), HIIT may produce comparable or even superior effects for certain outcomes, although the findings remain inconsistent (8, 9). Notably, earlier meta-analyses often combined prediabetes and T2D populations, whereas more recent evidence has focused more specifically on prediabetes; however, the relative advantages of different exercise modalities across glycaemic and lipid-related outcomes remain incompletely understood (8, 9). This suggests that the potential value of HIIT may not be uniform across all outcomes, but may instead be outcome-specific.

Importantly, prediabetes itself is a heterogeneous condition that typically includes impaired fasting glucose (IFG), impaired glucose tolerance (IGT), and combinations of both. These subtypes differ in their underlying pathophysiology, future risk of progression to T2D, and cardiovascular risk profile, and individuals may also differ in their glycaemic and cardiometabolic responses to exercise intervention (4, 10). In addition, previous studies suggest that individuals with prediabetes may exhibit distinct pathophysiological features across age groups, further supporting the view that this population should not be regarded as a single homogeneous group (11). From this perspective, fasting blood glucose (FBG) and 2-hour plasma glucose (2hPG) may reflect different dimensions of dysglycaemia, and their responses to exercise intervention may therefore not be identical (10, 11). Accordingly, focusing on only one glycaemic indicator may be insufficient to fully capture the true effects of HIIT in adults with prediabetes. A more informative approach may be to examine FBG, 2hPG, and related cardiometabolic risk factors jointly, from the perspective of glycaemic phenotype differences and outcome-specific effects, in order to better identify the relative advantages of different exercise modalities and improve the precision of exercise prescription.

Although research on the metabolic benefits of HIIT has increased in recent years, several important gaps remain in the evidence base for adults with prediabetes. Existing studies show marked heterogeneity in participant characteristics, training protocols, comparator conditions, and outcome coverage, which limits the comparability, synthesis, and translation of the available evidence. At the same time, the relative effects of HIIT versus non-exercise controls or other exercise interventions in adults with prediabetes, particularly whether HIIT shows differential advantages across glycaemic outcomes such as FBG and 2hPG, have not been sufficiently synthesized and quantitatively evaluated in a focused manner (8, 9). Therefore, a more systematic evaluation of the effects of HIIT on glycaemic control and cardiometabolic risk factors in a prediabetes-specific population is warranted.

Accordingly, the present study conducted a systematic review and meta-analysis to compare the effects of HIIT with control conditions (non-exercise controls or other exercise interventions) on glycaemic control and cardiometabolic risk factors in adults with prediabetes. Glycaemic outcomes were defined as the primary outcomes, and cardiometabolic risk-related indicators were defined as the secondary outcomes. Unlike previous reviews that combined prediabetes with T2D or focused only on overall metabolic outcomes, this review specifically targeted adults with prediabetes, emphasized the differential patterns across glycaemic outcomes, particularly FBG and 2hPG, and interpreted the potential benefits of HIIT from an outcome-specific perspective. In doing so, this review aimed to provide evidence to inform exercise prescription and cardiometabolic risk management in adults with prediabetes.

## 2 Methods

### 2.1 Protocol registration

This review was registered in PROSPERO (CRD42024605154) (12). Prior to revision, the registration record was updated to ensure consistency with the study timeline reported in the present manuscript.

### 2.2 Literature search and study selection

#### 2.2.1 Information sources and search strategy

A systematic search was conducted in PubMed, Embase, Web of Science, EBSCO, and the Cochrane Library from database inception to January 2026. The search was restricted to studies published in English. The search strategy was developed around two core concepts, namely “prediabetes/Prediabetic State” and “high-intensity interval training,” and was expanded using both controlled vocabulary terms (e.g., MeSH in PubMed) and free-text terms adapted for each database.

Search terms related to prediabetes included prediabetes, prediabetic state, impaired glucose tolerance, impaired fasting glucose, IFG, IGT, non-diabetic hyperglycaemia, and intermediate hyperglycaemia, together with several additional terms related to high-risk states for diabetes. Search terms related to HIIT included high-intensity interval training, HIIT, high-intensity interval exercise, HIIE, high-intensity training, HIT, reduced-exertion high-intensity interval training, REHIT, sprint interval training, SIT, low-volume high-intensity interval training, LVHIIT*, and* high-volume high-intensity interval training, HVHIIT. The full search strategies for all databases are provided in **Supplementary Table S1**.

In addition to the electronic database search, backward citation searching of the reference lists of included studies and relevant reviews was performed to identify potentially missed studies. Two reviewers (Jun Li and Jin Yuan) independently performed title/abstract screening, full-text assessment, and final study selection. Disagreements were resolved through discussion, and when necessary, a third reviewer (Haifeng Ma) acted as arbiter. The study selection process was presented using a PRISMA flow diagram.

#### 2.2.2 Inclusion criteria

Eligibility criteria were defined according to the PICOS framework (participants, interventions, comparators, outcomes, and study design), as follows:

1. Participants (P): Adults aged ≥18 years with prediabetes. Prediabetes was defined according to the recognized diagnostic criteria used in the original studies, including impaired fasting glucose, impaired glucose tolerance, or other states explicitly defined by the study authors as prediabetes.
2. Intervention (I): Structured HIIT, defined as interval exercise protocols that included pre-specified high-intensity work bouts and recovery periods, with clear reporting of key training characteristics such as exercise intensity, interval structure, session duration, frequency, or intervention period.
3. Comparators (C): A non-exercise control (CON) or another exercise intervention comparator, such as MICT.
4. Outcomes (O): The primary outcomes were glycaemic control measures, including fasting blood glucose/fasting plasma glucose (FBG/FPG), glycated hemoglobin (HbA1c), and 2hPG. Secondary outcomes were cardiometabolic risk-related indicators, including body mass index (BMI), cardiorespiratory fitness (VO_2_peak/VO_2_max), blood pressure, and blood lipids.
5. Study design (S): Interventional experimental studies, with priority given to randomized controlled trials. Crossover trials and other controlled interventional studies were also eligible, provided that independent outcome data could be extracted.

#### 2.2.3 Exclusion criteria

Based on the study objective and the PICOS framework, the following exclusion criteria were applied to improve consistency and comparability across the included studies:

1. Ineligible publication types: Reviews, systematic reviews, meta-analyses, case reports, case series, conference abstracts, dissertations/theses, study protocols, and other publications that did not report formal study results.
2. Ineligible populations: Individuals with diagnosed type 1 diabetes (T1D) or T2D, pregnant populations, and populations with severe diabetic complications or other serious comorbidities.
3. Ineligible interventions: Exercise protocols not meeting the definition of structured HIIT; studies in which HIIT was combined with other exercise, pharmacological, dietary, or psychological interventions and the independent effect of HIIT could not be isolated; and studies in which a CON was the primary treatment.
4. Ineligible outcome data: Studies that did not report any of the prespecified primary or secondary outcomes. Studies reporting relevant outcomes but providing incomplete data that could not be extracted or converted for quantitative synthesis were retained for qualitative synthesis only and were not included in the corresponding meta-analysis.
5. Other reasons: Duplicate publications, studies for which the full text could not be retrieved, and secondary analyses of previously published studies that did not provide independent usable data.

### 2.3 Data extraction

Data extraction was performed independently by two reviewers (Jun Li and Jin Yuan). Any disagreements were resolved through discussion, and when necessary, a third reviewer (Haifeng Ma) acted as arbiter. A predesigned standardized data extraction form (Microsoft Excel) was used to collect the following information:

1. General study information: first author, year of publication, country or region, and study design;
2. Participant characteristics: sample size, sex, age, baseline BMI, and the diagnostic criteria or definition used for prediabetes;
3. Covariates and contextual control information: dietary control, medication use, baseline physical activity level, and other relevant background factors that might influence the interpretation of glycaemic and cardiometabolic outcomes;
4. Intervention characteristics: type of intervention, exercise modality, intensity, frequency, session duration, total intervention length, and key prescription parameters related to the FITT principle;
5. Comparator characteristics: comparator group type (non-exercise control or another exercise intervention) and its main intervention content;
6. Outcome measures: the primary outcomes were indicators of glycaemic control, including FBG/FPG, 2hPG, and HbA1c. Secondary outcomes were cardiometabolic risk-related indicators, including VO_2_peak/VO_2_max, BMI, blood pressure, and blood lipids;
7. Statistical data: mean and standard deviation (mean ± SD) at baseline and post-intervention for each outcome, together with any additional statistical information required for effect size calculation.

For all continuous outcomes, change-from-baseline values were preferentially extracted for meta-analysis. If change values were not directly reported in the original study, baseline and post-intervention means and standard deviations were extracted, and conversions or estimations were performed, where possible, in accordance with the recommendations of the Cochrane Handbook.

When glycaemic outcomes were reported in mg/dL, values were converted to mmol/L using the formula mg/dL ÷ 18. For multi-arm trials in which more than one HIIT intervention arm shared the same comparator group, the sample size of the shared comparator group was divided equally across the relevant intervention arms, in accordance with Cochrane Handbook guidance, to avoid unit-of-analysis errors due to double counting. If multiple post-intervention time points were reported, the time point corresponding to the prespecified end of the intervention was preferentially selected for the primary analysis. When reported units, statistical formats, or outcome definitions differed across studies, both reviewers cross-checked the data and standardized them before synthesis to ensure comparability across studies.

### 2.4 Quality assessment

Risk of bias in the included studies was independently assessed by two reviewers (Jun Li and Jin Yuan). Disagreements were resolved through discussion, and when necessary, a third reviewer (Haifeng Ma) acted as arbiter.

For included randomized controlled trials (RCTs), the Cochrane Risk of Bias 2 (RoB 2) tool was used to assess bias across the following domains: bias arising from the randomization process, bias due to deviations from intended interventions, bias due to missing outcome data, bias in outcome measurement, and bias in selection of the reported result. Each domain, as well as the overall risk of bias, was judged as “low risk,” “some concerns,” or “high risk”.

No numerical summary score was assigned for risk of bias. Instead, the methodological quality of the included studies was presented using domain-level judgments together with an overall judgment. The certainty of evidence was evaluated using the GRADE approach for the primary outcomes and key secondary outcomes included in the quantitative synthesis. Evidence was rated across five domains: risk of bias, inconsistency, indirectness, imprecision, and publication bias, and was categorized as high, moderate, low, or very low (13).

### 2.5 Statistical analysis

Meta-analyses were primarily conducted using Review Manager (RevMan) 5.3, with additional calculations and sensitivity analyses performed when required according to the Cochrane Handbook. Effect sizes were pooled using the inverse-variance method. Glycemic control outcomes and cardiometabolic risk-related outcomes were synthesized separately. For continuous outcomes, mean differences (MDs) with 95% confidence intervals (95% CIs) were used when studies reported outcomes on the same scale. When the same outcome was assessed using different scales or measurement methods across studies, standardized mean differences (SMDs) with 95% CIs were used. For outcomes pooled as SMDs, Hedges’ g was applied to correct for small-sample bias.

Both primary and secondary outcomes were preferentially pooled using change-from-baseline values. For parallel-group trials, the between-group effect size was defined as the difference between the change in the HIIT group and the change in the comparator group. When the standard deviation of the change score (SD_change) was not directly reported, it was estimated from baseline and post-intervention standard deviations according to the recommendations of the Cochrane Handbook, using the following formula:


$$SD_{change}=\sqrt{SD_{baseline}^{2}+SD_{final}^{2}-2r\times SD_{baseline}\times SD_{final}}$$

The primary analyses assumed a correlation coefficient of r, 0.50. Sensitivity analyses were additionally performed using r, 0.25 and r, 0.75 to assess the robustness of the pooled estimates to the assumed correlation coefficient.

Between-study heterogeneity was assessed using Cochran’s Q test and the I² statistic. In general, when I² ≤ 50%, statistical heterogeneity was considered low and a fixed-effect model was preferred. When I² > 50%, or when substantial clinical or methodological heterogeneity was judged to be present based on study design, intervention characteristics, or outcome measurement, a random-effects model was used. The interpretation of heterogeneity was based on both statistical results and clinical context rather than on a single threshold alone. For outcomes with substantial heterogeneity, interpretation focused particularly on intervention duration, differences in HIIT protocols, and baseline participant characteristics such as age and BMI.

Leave-one-out sensitivity analysis was performed by sequentially removing one study at a time and repeating the meta-analysis to assess the influence of individual studies on the overall pooled effect and heterogeneity estimates. For multi-arm trials in which multiple HIIT intervention arms shared a single comparator group, the sample size of the shared comparator group was divided equally across the relevant intervention arms in accordance with the Cochrane Handbook, in order to avoid unit-of-analysis errors caused by double counting.

Publication bias was assessed qualitatively using funnel plots only when at least 10 studies were available for a given outcome. Given the limited number of included studies and the potential influence of between-study heterogeneity on funnel plot interpretation, formal assessment of publication bias was not performed when too few studies were available; instead, its possible presence was considered cautiously in the interpretation of the findings.

Exploratory subgroup analyses were considered when a given outcome included a sufficient number of studies and the original reports provided adequate information, particularly when substantial heterogeneity was present (I² > 50%). Prespecified subgroup variables included intervention duration, participant age, sex, and BMI. However, whether subgroup analyses were undertaken depended on the number of available studies and the completeness and comparability of the reported data. Because dietary control, medication use, and baseline physical activity were inconsistently reported across the original studies and the completeness of these data was limited, these variables were not included in quantitative subgroup analyses; instead, their potential influence was considered cautiously in the interpretation and discussion of the findings. All tests were two-sided, and P < 0.05 was considered statistically significant.

## 3 Results

### 3.1 Literature search results

A total of 2,640 records were initially identified through database searching, including 194 from EBSCO, 606 from Embase, 534 from Web of Science, 86 from PubMed, and 1,220 from the Cochrane Library. After duplicate removal in EndNote, 722 duplicate records were excluded, leaving 1,918 records for title screening. Following title screening, 1,874 records were excluded, mainly because they were not relevant to the topic or were reviews, systematic reviews/meta-analyses, or study protocols. Forty-four records were retained for abstract screening.

**Figure 1:**
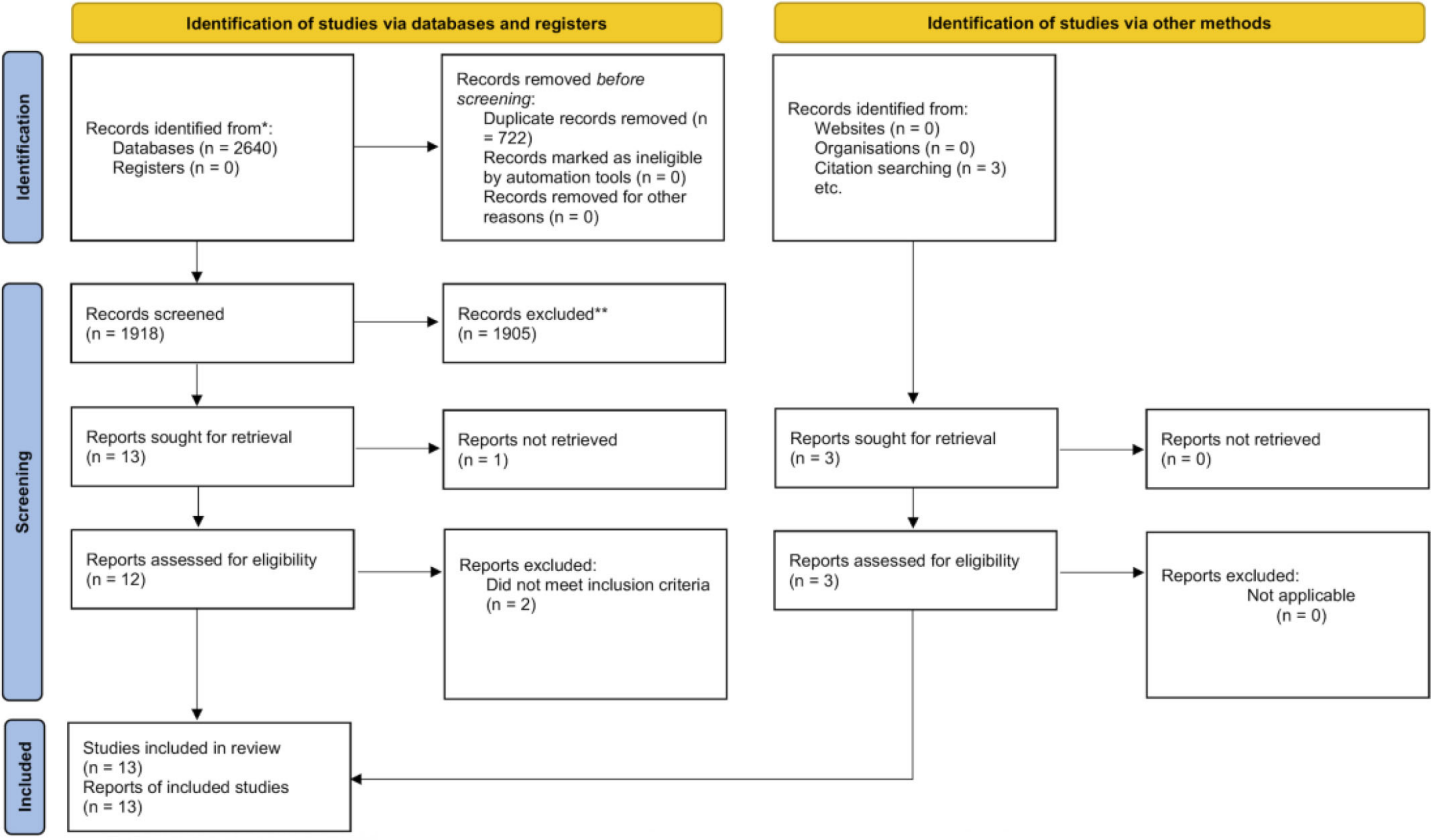
Flow diagram of study selection.

After abstract screening, a further 31 records were excluded for not meeting the eligibility criteria, leaving 13 reports for full-text assessment. Of these, 3 reports were excluded after full-text review, including 1 for failure to retrieve the full text and 2 for not meeting the inclusion criteria. Subsequently, 3 additional studies were identified through backward citation searching of included studies and related reviews. In total, 13 studies were included in the review. The study selection process is presented in **Figure 1**.

### 3.2 Risk of bias assessment

The 13 included RCTs were assessed using the Cochrane RoB 2 tool. Overall, most studies were judged as having some concerns, whereas a small number were judged as being at high risk of bias. No study was judged as having an overall low risk of bias.

For bias arising from the randomization process, only a small number of studies clearly reported both the generation of the random sequence and allocation concealment and were therefore judged as low risk. In contrast, most studies only stated that participants were “randomized” or “block randomized” without sufficient detail regarding sequence generation or allocation concealment, and were therefore judged as having some concerns.

For bias due to deviations from intended interventions, this domain was one of the main contributors to judgments of some concerns, because blinding of participants and intervention providers is generally difficult in exercise trials, and several studies did not clearly report whether intention-to-treat analyses were used or instead relied on per-protocol or completer analyses. A few studies were judged as high risk in this domain.

Bias in outcome measurement was generally low, largely because most outcomes, including fasting glucose, 2-hour glucose, HbA1c, BMI, blood pressure, and VO_2_peak, were objectively measured. For bias in selection of the reported result, most studies were judged as having some concerns because study protocols or prespecified statistical analysis plans were not publicly available. A few studies were judged as high risk because of limited reporting transparency or inconsistencies across different reporting sources.

**Figure 2:**
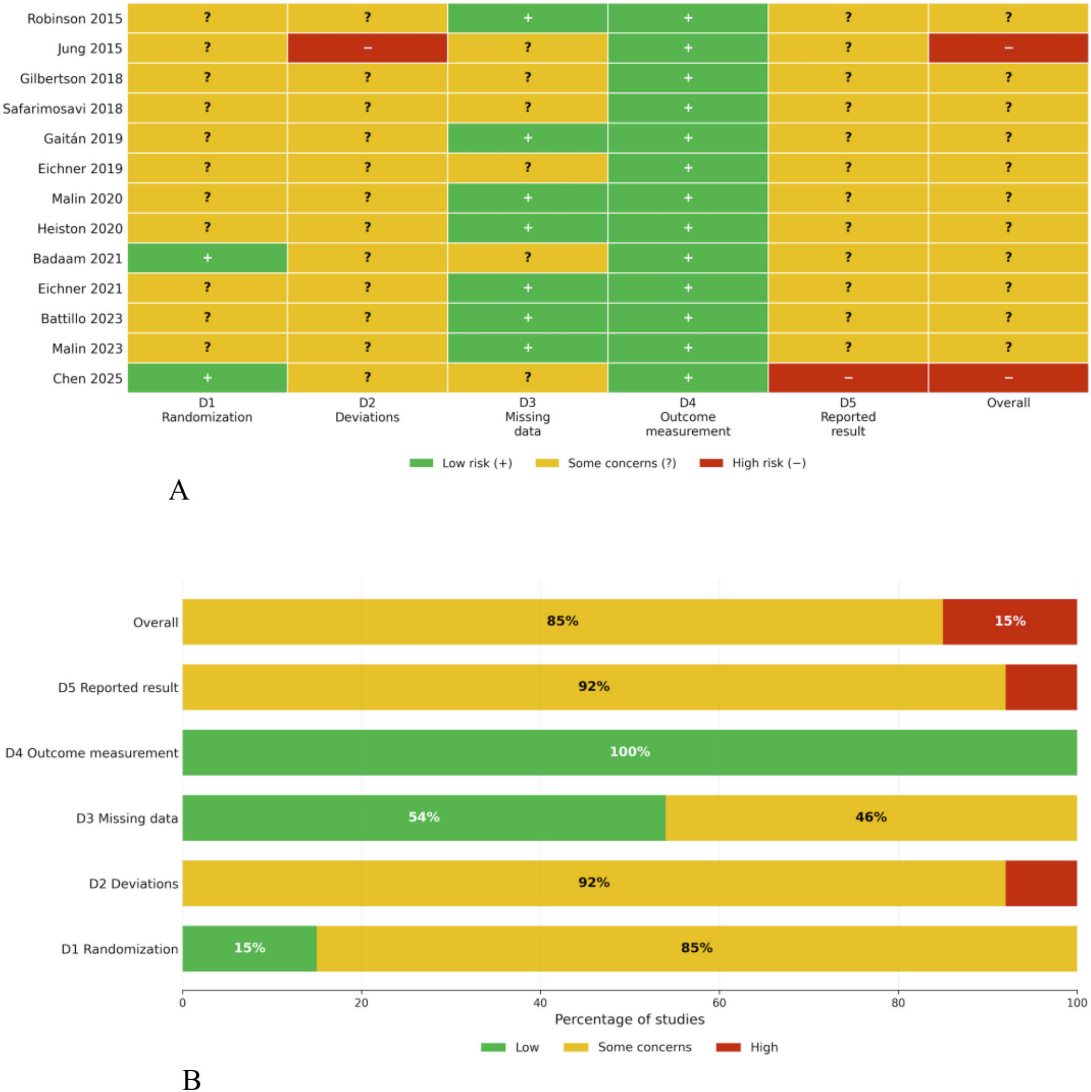
**(A)** RoB 2 traffic-light plot for the included randomized controlled trials. **(B)** RoB 2 summary plot for the included randomized controlled trials.

Overall, the main sources of bias among the included studies were insufficient reporting of the randomization process, inadequate handling of deviations from intended interventions, and limited transparency in outcome reporting, whereas the risk of bias related to objective outcome measurement was generally low. Domain-level judgments and overall patterns are shown in **Figures 2A, B**.

### 3.3 Study characteristics

#### 3.3.1 Participant characteristics

A total of 13 independent randomized controlled trials were included. Overall, most studies were small-sample exercise interventions, and only a few studies included at least 20 participants in each group. The study populations were predominantly middle-aged or older adults who were overweight or obese, although a small number of studies included younger adults with prediabetes or participants with relatively lower BMI. Participant characteristics of the included studies are presented in **Table 1**.

**Table 1:** Participant characteristics of the included studies.

Author	Year	Group	n	Male/female	Age (years)	BMI (kg/m²)
Robinson et al.	2015	HIIT	20	3/17	52 ± 10	32.9 ± 6.6
		MICT	19	4/15	52 ± 10	31.4 ± 4.1
Jung et al.	2015	HIIT	15	4/11	51 ± 11	33.1 ± 7.7
		MICT	17	1/16	51 ± 10	32.8 ± 5.0
Gilbertson et al.	2018	HIIT	14	3/11	60.4 ± 2.0	32.1 ± 1.3
		MICT	17	4/13	61.3 ± 2.1	34.5 ± 1.6
Safarimosavi et al.	2018	HIIT	8	NR	38.6 ± 4.5	27.3 ± 3.3
		CON	8	NR	37.4 ± 3.2	27.0 ± 2.2
Gaitán et al.	2019	HIIT	11	2/9	60.1 ± 2.3	32.7 ± 1.6
		MICT	11	3/8	65.6 ± 1.9	31.8 ± 1.8
Eichner et al.	2019	HIIT	14	3/11	60.4 ± 2.0	32.1 ± 1.3
		MICT	14	3/11	62.1 ± 2.2	34.5 ± 1.9
Malin et al.	2020	HIIT	12	3/9	59.9 ± 2.2	30.9 ± 1.1
		MICT	14	3/11	60.4 ± 2.3	35.6 ± 1.6
Heiston et al.	2020	HIIT	14	3/11	60.0 ± 7.6	32.0 ± 5.0
		MICT	14	3/11	61.0 ± 9.4	34.0 ± 5.9
Badaam et al.	2021	HIIT	74	NR	31.0 ± 3.4	28.6 ± 3.1
		MICT	72	NR	30.7 ± 3.3	28.2 ± 2.9
Eichner et al.	2021	HIIT	10	2/8	61.7 ± 1.9	30.9 ± 1.4
		MICT	8	2/6	67.4 ± 2.0	31.3 ± 2.6
Battillo et al.	2023	HIIT	14	3/11	60.4 ± 2.0	32.1 ± 1.3
		MICT	14	3/11	62.1 ± 2.2	34.5 ± 1.9
Malin et al.	2023	HIIT	11	2/9	60.3 ± 2.4	32.1 ± 1.2
		MICT	12	2/10	60.8 ± 2.4	34.0 ± 1.7
Chen et al.	2025	HIIT	29	17/12	51.0 ± 4.4	23.8 ± 1.4
		MICT	36	19/17	51.7 ± 4.6	24.7 ± 2.0

HIIT, high-intensity interval training; MICT, moderate-intensity continuous training; CON, control group; BMI, body mass index; NR, not reported.

#### 3.3.2 Inclusion criteria and outcomes

Across the included studies, prediabetes was generally defined on the basis of the American Diabetes Association (ADA) criteria or other recognized diagnostic criteria adopted in the original studies, primarily using FBG/FPG, 2hPG after a 75 g oral glucose tolerance test, and/or HbA1c. In a small number of studies, additional eligibility criteria were applied alongside glycaemic indices, such as diabetes risk questionnaires, BMI thresholds, prior physician diagnosis, or sedentary lifestyle, in order to improve identification of the target population.

In terms of outcome reporting, the included studies mainly focused on glycaemic control and cardiometabolic risk-related indicators. The primary outcomes included FBG/FPG, 2hPG, and HbA1c. Secondary outcomes mainly included BMI, VO_2_peak/VO_2_max, blood pressure, and blood lipids. Overall, BMI and VO_2_peak/VO_2_max were the most frequently reported secondary outcomes, whereas HbA1c, blood pressure, and blood lipids were reported in only a subset of studies. The key inclusion criteria of each study and the primary/secondary outcomes extractable for the present review are summarized in **Table 2**. More detailed information is provided in **Supplementary Table S2**.

**Table 2:** Inclusion criteria and extractable outcomes of the included studies.

Author	Year	Key inclusion criteria	Extractable outcomes in the present review
Robinson et al.	2015	HbA1c (ADA) ①②④	FBG; BMI, VO_2_peak, blood pressure
Jung et al.	2015	FBG/HbA1c (ADA) ①③	BMI, VO_2_peak, blood pressure
Gilbertson et al.	2018	OGTT, HbA1c (ADA)	2hPG; BMI, VO_2_peak
Safarimosavi et al.	2018	FBG/OGTT (original study criteria)	FBG, 2hPG, HbA1c
Gaitán et al.	2019	OGTT (ADA)	FBG, 2hPG; BMI, VO_2_peak
Eichner et al.	2019	OGTT (ADA)	BMI, VO_2_peak, blood pressure
Malin et al.	2020	FBG, OGTT (ADA)	FBG, 2hPG; BMI, VO_2_peak
Heiston et al.	2020	FBG, OGTT (ADA) ②	FBG, 2hPG; BMI, VO_2_peak, blood pressure, lipid profile
Badaam et al.	2021	FBG, OGTT (ADA)	FBG, HbA1c; BMI
Eichner et al.	2021	OGTT (ADA)	FBG, 2hPG; BMI, VO_2_peak
Battillo et al.	2023	FBG, OGTT, HbA1c (ADA)	BMI, VO_2_peak, blood pressure, lipid profile
Malin et al.	2023	FBG, OGTT (ADA)	FBG, 2hPG; BMI, VO_2_peak
Chen et al.	2025	FBG, 2hPG, HbA1c (original study criteria)	FBG, 2hPG, HbA1c; BMI, VO_2_peak, blood pressure, lipid profile

① diabetes risk questionnaire; ② BMI criterion; ③ physician diagnosis; ④ sedentary lifestyle. ADA, American Diabetes Association; OGTT, oral glucose tolerance test; FBG, fasting blood glucose; 2hPG, 2-hour postprandial glucose; HbA1c, glycated hemoglobin; BMI, body mass index; VO_2_peak, peak oxygen uptake.

#### 3.3.3 Intervention characteristics

Among the 13 included studies, 1 compared HIIT with a CON, whereas the remaining 12 compared HIIT with MICT. Intervention duration ranged from 13 days to 12 weeks. Of these, 9 studies had intervention periods of 2 weeks or less, whereas 4 studies lasted longer than 2 weeks. Cycle ergometer training was the predominant exercise modality, although a small number of studies used walking, treadmill exercise, or self-selected aerobic exercise.

Overall, the HIIT protocols could be broadly categorized into three main formats: 10 × 3-min high-intensity intervals interspersed with 3-min active recovery, 10 × 1-min high-intensity intervals interspersed with 1-min recovery, and 4 × 1-min all-out sprints interspersed with 90-s recovery. In contrast, the MICT protocols mainly consisted of continuous moderate-intensity aerobic exercise, with session duration generally ranging from 20 to 60 min and exercise intensity most commonly prescribed at approximately 55%–70%HRpeak/HRmax. More detailed information is provided in **Supplementary Table S3**. In addition, to further characterize differences in intervention context and related control conditions across studies, supplementary information on dietary control, medication use, baseline physical activity, and other relevant background factors is summarized in **Supplementary Table S4**.

### 3.4 Outcome analyses

#### 3.4.1 Glycemic control

##### 3.4.1.1 HbA1c (%)

For the comparison between HIIT and the CON, only one study was available and no meta-analysis was performed. That study showed a downward trend in HbA1c after HIIT, whereas HbA1c increased slightly in the CON group (14).

**Figure 3:**
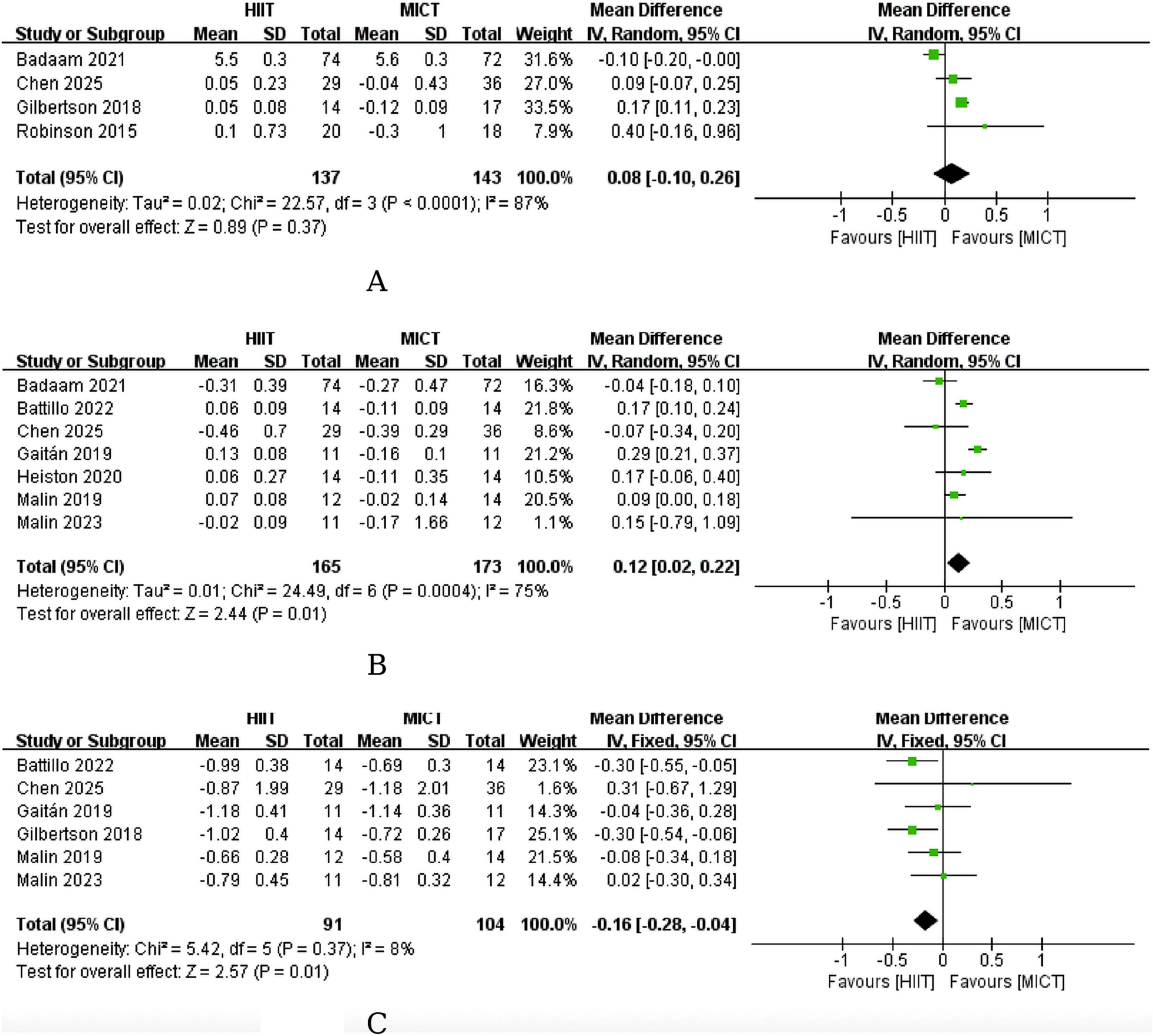
**(A)** Effect of HIIT versus MICT on HbA1c. **(B)** Effect of HIIT versus MICT on HbA1c. **(C)** Effect of HIIT versus MICT on 2-hour plasma glucose.

For the comparison between HIIT and MICT, four studies were included in the meta-analysis. The pooled effect estimate was MD, 0.08% (95% CI: −0.10 to 0.26, P, 0.37), indicating no significant between-group difference in HbA1c improvement. Between-study heterogeneity was high (I², 87%), and the directions of the individual study effects were inconsistent, with one study favoring HIIT and three studies favoring MICT. Therefore, the current evidence is insufficient to support the superiority of HIIT over MICT in improving HbA1c in adults with prediabetes. The results are shown in **Figure 3A**. According to GRADE, the certainty of evidence for this outcome was very low (**Supplementary Table S5**).

##### 3.4.1.2 FBG (mmol/L)

For the comparison between HIIT and CON, only one study was available and no meta-analysis was performed. That study showed that FBG decreased from baseline in the HIIT group, whereas it increased from baseline in the CON group (14), suggesting that HIIT may help improve fasting glycemic status in adults with prediabetes.

For the comparison between HIIT and MICT, seven studies were included in the meta-analysis, and effect estimates were pooled using change-from-baseline values. Because heterogeneity was substantial (I², 75%), the primary analysis used a random-effects model. The pooled result showed a statistically significant difference between groups (MD, 0.12 mmol/L, 95% CI: 0.02 to 0.22, P, 0.01), indicating a small but statistically significant advantage of MICT over HIIT in reducing FBG under the current body of evidence (**Figure 3B**). This finding suggests that the effects of different exercise modalities on fasting glucose-related phenotypes may not be uniform.

Sensitivity analysis indicated that this outcome was somewhat sensitive to individual studies. After sequential exclusion of each study, removal of Battillo et al. (2023) changed the overall statistical significance, while heterogeneity was not materially reduced. In contrast, removal of Gaitán et al. (2019) reduced heterogeneity, but the direction and statistical significance of the pooled effect remained unchanged. These findings suggest that the current evidence regarding the comparative effects of HIIT and MICT on FBG in adults with prediabetes has limited stability. According to GRADE, the certainty of evidence for this outcome was very low (**Supplementary Table S5**).

##### 3.4.1.3 2hPG (mmol/L)

For the comparison between HIIT and CON, only one study was available and no meta-analysis was performed. That study showed that 2hPG decreased from baseline in the HIIT group (−1.24 ± 0.64 mmol/L), whereas it increased from baseline in the CON group (0.20 ± 0.58 mmol/L) (14), suggesting that HIIT may help improve post-challenge glycemic control in adults with prediabetes.

For the comparison between HIIT and MICT, six studies were included in the meta-analysis. Pooled analysis based on change-from-baseline values using a fixed-effect model showed a statistically significant between-group difference (MD, −0.16 mmol/L, 95% CI: −0.28 to −0.04, P, 0.01), with the effect favoring HIIT. This result suggests that HIIT may be more beneficial than MICT for reducing 2hPG (**Figure 3C**).

In contrast to the findings for FBG, this result suggests that the effects of different exercise modalities on post-challenge glycemic phenotypes may differ. Sensitivity analysis showed that this outcome was also influenced by individual studies. After sequential removal of studies, exclusion of either Battillo et al. or Gilbertson et al. (2018) changed the overall statistical significance, while heterogeneity decreased further to 0%, suggesting that the primary result was influenced to some extent by individual studies. Because fewer than 10 studies were available, publication bias was not formally assessed using a funnel plot according to the prespecified criterion. According to GRADE, the certainty of evidence for this outcome was low (**Supplementary Table S5**); therefore, the finding should still be interpreted cautiously.

#### 3.4.2 Cardiorespiratory fitness

**Figure 4:**
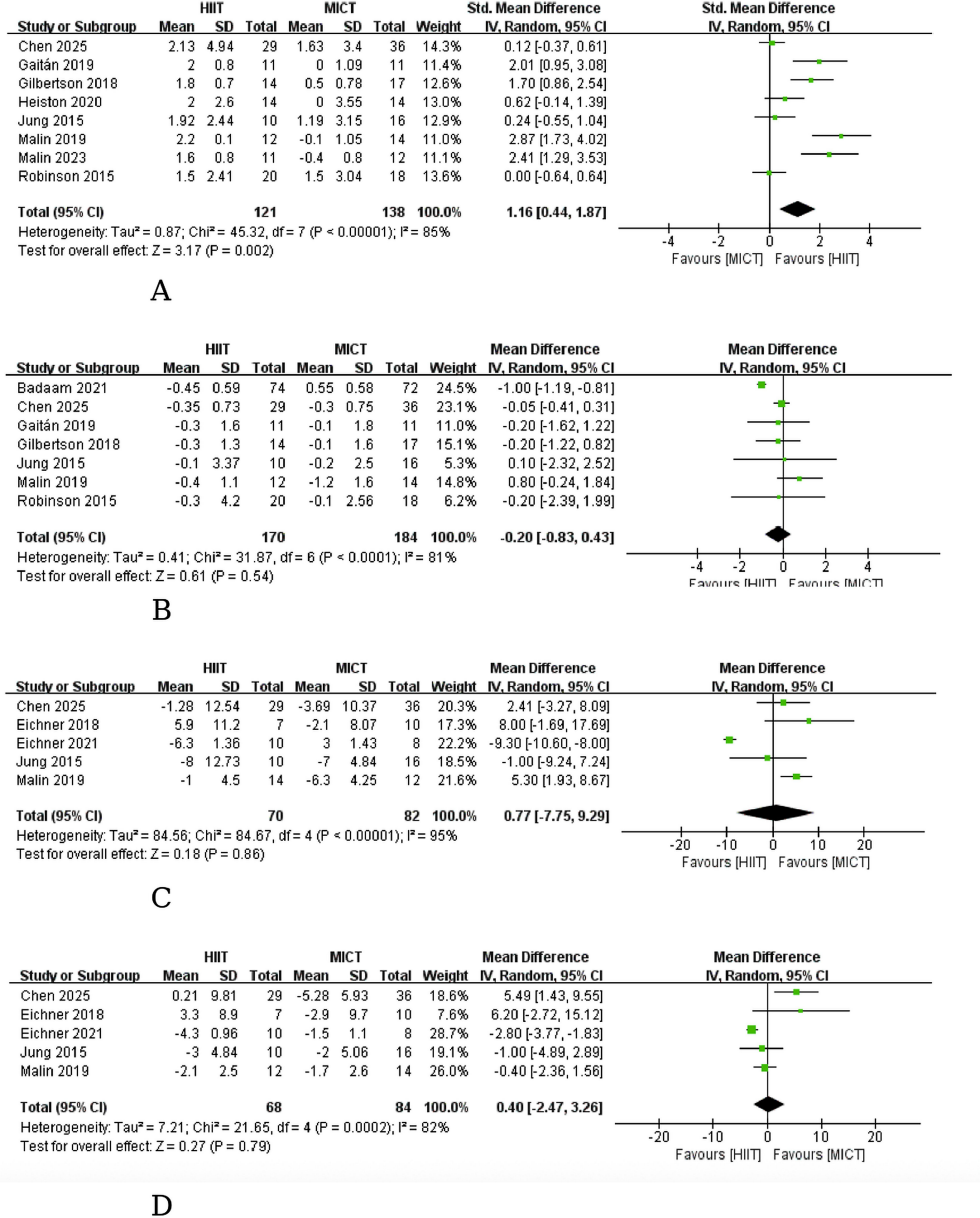
**(A)** Effects of HIIT versus MICT on cardiorespiratory fitness (VO_2_peak/VO_2_max). **(B)** Effect of HIIT versus MICT on body mass index. **(C)** Effects of HIIT versus MICT on SBP. **(D)** Effects of HIIT versus MICT on DBP.

Cardiorespiratory fitness outcomes included VO_2_peak or VO_2_max (mL·kg^-^¹·min^-^¹). For the comparison between HIIT and the CON, no cardiorespiratory fitness data were available for quantitative synthesis. For the comparison between HIIT and MICT, eight studies were included in the meta-analysis. Effect sizes were pooled as standardized mean differences (SMDs, Hedges’ g) based on change-from-baseline values. Because heterogeneity was substantial (I², 85%), the primary analysis used a random-effects model. The pooled result showed that HIIT significantly improved cardiorespiratory fitness compared with MICT (SMD, 1.16, 95% CI: 0.44 to 1.87, P, 0.002), with the direction of effect favoring HIIT (**Figure 4A**).

Leave-one-out sensitivity analysis showed that the direction and statistical significance of the pooled effect remained unchanged after removal of any single study, and heterogeneity was not materially reduced, suggesting that the result for this outcome was relatively robust despite the presence of substantial between-study heterogeneity. Because the number of included studies was limited and did not meet the prespecified conditions for subgroup analysis, no subgroup analysis was performed. In addition, because fewer than 10 studies were available, publication bias was not formally assessed using a funnel plot. According to GRADE, the certainty of evidence for this outcome was moderate (**Supplementary Table S5**).

#### 3.4.3 Body composition

BMI, as an anthropometric outcome, was not available for quantitative synthesis in the comparison between HIIT and the CON. For the comparison between HIIT and MICT, seven studies were included in the meta-analysis. Pooled analysis based on change-from-baseline values showed no statistically significant difference between groups (MD, −0.20 kg/m², 95% CI: −0.83 to 0.43, P, 0.54), indicating that HIIT and MICT had broadly comparable effects on BMI. Because heterogeneity was substantial (I², 81%), the primary analysis used a random-effects model (**Figure 4B**).

Leave-one-out sensitivity analysis showed that, after exclusion of Badaam et al. (2021), heterogeneity decreased to 0%, whereas the direction and statistical significance of the pooled effect remained unchanged, suggesting that the heterogeneity for this outcome was mainly driven by an individual study. Because the number of included studies was limited and did not meet the prespecified conditions for subgroup analysis, no subgroup analysis was performed. In addition, because fewer than 10 studies were available, publication bias was not formally assessed using a funnel plot. According to GRADE, the certainty of evidence for this outcome was very low (**Supplementary Table S5**).

Given that changes in BMI usually require a longer intervention period to become stable and clinically evident, whereas most of the included studies were of short duration, the current finding is more likely to reflect short-term fluctuation or early adaptation rather than stable anthropometric change.

#### 3.4.4 Blood pressure

Blood pressure outcomes reported in the included studies comprised systolic blood pressure (SBP) and diastolic blood pressure (DBP), expressed in mmHg. For the comparison between HIIT and the CON, no blood pressure data were available for quantitative synthesis. For the comparison between HIIT and MICT, five studies were included in the meta-analyses of SBP and DBP, respectively. Pooled analyses based on change-from-baseline values showed no statistically significant between-group difference for either SBP (MD, 0.77 mmHg, 95% CI: −7.75 to 9.29, P, 0.86) or DBP (MD, 0.40 mmHg, 95% CI: −2.47 to 3.26, P, 0.79). Because heterogeneity was substantial for both outcomes (SBP: I², 95%; DBP: I², 82%), random-effects models were used in the primary analyses (**Figures 4C, D**).

Sensitivity analysis indicated that the SBP result was sensitive to individual studies. After exclusion of Eichner et al. (2021), between-study heterogeneity decreased markedly and the overall statistical conclusion changed, suggesting limited stability of the pooled result for SBP. In contrast, for DBP, sequential removal of individual studies did not materially alter the direction or statistical significance of the pooled effect, although heterogeneity remained high. The observed heterogeneity in blood pressure outcomes may be related to differences in intervention duration, variation in HIIT protocols, baseline blood pressure levels of the participants, and inconsistent reporting of dietary control and medication use across studies. These findings should therefore be interpreted with caution. According to GRADE, the certainty of evidence was very low for SBP and low for DBP (**Supplementary Table S5**).

#### 3.4.5 Blood lipids

Blood lipid outcomes included total cholesterol (TC), triglycerides (TG), high-density lipoprotein cholesterol (HDL-C), and low-density lipoprotein cholesterol (LDL-C), expressed in mmol/L. Because only a limited number of studies reported lipid outcomes, and the specific lipid variables and statistical reporting formats differed across studies, no meta-analysis could be performed. Therefore, these outcomes were synthesized narratively.

Overall, only a small subset of the included studies reported blood lipid-related outcomes. In comparisons between HIIT and MICT, the available evidence suggested that both exercise modalities may reduce TG, although in some individual studies the magnitude of TG reduction appeared greater with MICT. For TC, LDL-C, and HDL-C, most available studies did not observe clear between-group differences (15). Other studies suggested that both exercise modalities may improve selected lipid outcomes, although the findings were not consistent across studies (16).

Taken together, the current evidence does not clearly support a superiority of HIIT over MICT for improving the lipid profile in adults with prediabetes. Given the limited number of studies, the inconsistency in outcome reporting, and the absence of quantitative synthesis, no GRADE certainty assessment was performed for this outcome.

## 4 Discussion

This systematic review and meta-analysis synthesized intervention studies investigating the effects of HIIT in adults with prediabetes, with a particular focus on glycemic control and cardiometabolic risk-related outcomes. Unlike previous reviews that either combined prediabetes with T2D or primarily compared the overall metabolic effects of HIIT and MICT, the present review specifically focused on adults with prediabetes and further highlighted the differential findings for FBG and 2hPG, thereby emphasizing that different glycemic phenotypes may respond differently to exercise stimuli. Overall, the current evidence suggests that HIIT, as a time-efficient exercise strategy, has potential utility during this critical intervention window in prediabetes; however, its comparative advantages over MICT appear to be clearly outcome-specific.

Specifically, the comparative findings for glycemic outcomes were not uniform. The pooled analysis for FBG suggested that MICT may be more favorable for lowering fasting glucose, whereas the pooled analysis for 2hPG suggested that HIIT may be more beneficial for improving post-challenge glycemic control. For HbA1c, no statistically significant difference was observed between HIIT and MICT, and the available evidence was derived from only a small number of studies. In contrast, HIIT showed a more consistent positive effect on cardiorespiratory fitness, with a statistically significant advantage for improving VO_2_peak/VO_2_max and moderate certainty of evidence for this outcome. By comparison, BMI, blood pressure, and blood lipid outcomes did not show clear evidence of superiority of HIIT over MICT. Importantly, most included interventions were of short duration, often no longer than 2 weeks, meaning that some of the observed changes are more likely to reflect acute physiological responses or early adaptation rather than stable clinical benefit.

From a methodological perspective, all included studies were randomized controlled trials; however, most were judged as having some concerns regarding the randomization process, deviations from intended interventions, and transparency of outcome reporting. Owing to the nature of exercise intervention studies, strict double blinding and placebo-controlled designs are difficult to implement in practice. In addition, small sample sizes, short intervention duration, and incomplete reporting of outcomes in several studies contributed to low certainty of evidence for some glycemic and cardiometabolic outcomes. At this stage, the findings are therefore better interpreted as indicating that HIIT may exert differential effects across metabolic outcomes in adults with prediabetes, with more promising signals for improving cardiorespiratory fitness and selected post-challenge glycemic outcomes, while evidence for broader benefits on overall glycemic control and cardiometabolic risk remains insufficient and requires confirmation in larger, better-designed, and more comprehensively reported randomized controlled trials.

### 4.1 Effects on glycemic outcomes

#### 4.1.1 HbA1c

HbA1c is a commonly used integrative indicator of glycemic control in clinical practice, reflecting average glycemic exposure over approximately 2–3 months, and is closely associated with diabetes-related complications and cardiovascular risk (17, 18).

For the comparison between HIIT and the CON, only one study was available, and this study suggested that HbA1c tended to decrease after HIIT, whereas it increased slightly in the CON group, indicating that HIIT may help improve intermediate-term glycemic control in adults with prediabetes (14). However, in the comparison between HIIT and MICT, the meta-analysis showed no statistically significant difference in HbA1c improvement between the two exercise modalities, and between-study heterogeneity was high. This finding is not entirely consistent with some previous studies in people with T2D, in which HIIT was reported to reduce HbA1c, and it further suggests that conclusions derived from T2D populations may not be directly generalizable to adults with prediabetes, particularly in the context of short intervention duration and greater heterogeneity in glycemic phenotype (19–22).

A more plausible explanation for this discrepancy is, first, that HbA1c is highly sensitive to intervention duration. Most of the trials included in the present review were short-term interventions, with several lasting only about 2 weeks, which is unlikely to adequately capture the longer time frame reflected by HbA1c. As such, HbA1c may be relatively insensitive to short-term exercise adaptation. Second, marked differences across studies in participant age, degree of adiposity, baseline glycemic phenotype, and exercise protocol may also have contributed to the inconsistent direction of change in HbA1c. Taken together with the existing evidence, the more cautious interpretation at present is that HIIT may have some potential to improve HbA1c, but there is currently no stable evidence supporting an additional advantage of HIIT over MICT for HbA1c in adults with prediabetes. Further randomized controlled trials with longer intervention periods and more standardized outcome reporting are needed to clarify the true effects of different exercise modalities on HbA1c.

#### 4.1.2 FBG

FBG primarily reflects metabolic features related to IFG, including hepatic glucose output, basal insulin resistance, and impaired β-cell function (11). In the present meta-analysis, MICT appeared to be more favorable than HIIT for lowering FBG; however, this finding was accompanied by substantial heterogeneity and very low certainty of evidence, and should therefore be interpreted cautiously. This result is not entirely consistent with previous meta-analyses in T2D populations, in which no significant difference between HIIT and MICT was observed for FBG improvement (19, 23). One possible explanation is that improvement in FBG may depend, at least in part, more strongly on accumulated exercise volume, continuous exercise duration, and related energy expenditure, for which MICT may be better suited because of its longer uninterrupted training duration and its potentially greater influence on basal glucose homeostasis and hepatic glucose metabolism (24).

At the same time, the included trials differed considerably in participant composition, glycemic phenotype, intervention duration, and control of co-interventions. Sensitivity analysis also indicated that this outcome was influenced by individual studies. Therefore, the current evidence is better interpreted as suggesting that MICT may hold a slight advantage over HIIT for FBG, but that this conclusion is not yet robust enough to support a definitive inference. Future studies should distinguish more clearly between IFG, IGT, and mixed prediabetes phenotypes at the design stage, and should report co-intervention factors such as diet, medication use, and habitual physical activity more comprehensively in order to improve the interpretability of evidence for FBG.

#### 4.1.3 2hPG

2hPG more directly reflects IGT and metabolic abnormalities related to peripheral insulin resistance, particularly in skeletal muscle (25). Previous studies have suggested that aerobic exercise can improve 2hPG in adults with prediabetes (26, 27). In the present review, the meta-analysis comparing HIIT and MICT showed that HIIT may be more favorable than MICT for reducing 2hPG, and between-study heterogeneity was low. In contrast to the findings for FBG, this result suggests that responses of different glycemic outcomes to exercise may be clearly outcome-specific. When considered together, the FBG and 2hPG findings support the possibility that different glycemic phenotypes may respond differently to exercise stimuli. Compared with fasting glucose, which is more closely related to basal glucose homeostasis and hepatic glucose output, post-challenge glucose may be more sensitive to high-intensity intermittent exercise stimuli.

From a mechanistic perspective, HIIT may more effectively stimulate skeletal muscle glucose uptake, improve peripheral insulin sensitivity, and enhance post-exercise glucose disposal through repeated bouts of high-intensity work, thereby offering a potential advantage for post-challenge glycemic regulation. In contrast, FBG is more strongly influenced by hepatic glucose output and basal metabolic regulation, and therefore may not show the same direction of training response as 2hPG. Nevertheless, the certainty of evidence for 2hPG remained low, and sensitivity analysis indicated that the primary result was influenced to some extent by individual studies. Accordingly, the more appropriate conclusion at this stage is that HIIT shows a promising signal for improving 2hPG in adults with prediabetes, but this finding still requires confirmation in more high-quality trials. Future studies should strengthen standardization of training intensity, interval structure, total exercise dose, and lifestyle co-interventions in order to clarify more precisely the true effects of different exercise modalities on post-challenge glycemic regulation.

### 4.2 Effects on cardiometabolic risk-related outcomes

#### 4.2.1 Cardiorespiratory fitness (VO_2_peak/VO_2_max)

VO_2_peak and VO_2_max are core indicators of cardiorespiratory fitness, and higher levels are closely associated with lower risks of cardiovascular disease, incident type 2 diabetes, and related mortality (8). In the present meta-analysis, HIIT showed a statistically significant advantage over MICT for improving cardiorespiratory fitness, suggesting that HIIT may have considerable practical potential for enhancing cardiorespiratory fitness in adults with prediabetes. This finding is broadly consistent with previous literature (28). Given that most included participants were overweight or obese, and that some study populations were older, the greater metabolic stimulus provided by HIIT may be more favorable for eliciting both peripheral oxygen utilization and central cardiopulmonary adaptations, thereby producing larger improvements in VO_2_peak/VO_2_max within a relatively short period.

It should be noted, however, that although the direction of the pooled effect for this outcome was relatively consistent, between-study heterogeneity remained high, indicating substantial differences across studies in participant characteristics, training modality, intervention duration, and outcome measurement. Previous research suggests that regular exercise may induce relatively rapid cardiorespiratory adaptation during the early phase of training, whereas the magnitude of improvement may gradually plateau with longer intervention duration (29). This may partly explain why some short-term intervention studies reported relatively large improvements in VO_2_.

In addition, previous work has shown that, under comparable training intensity and frequency, low-volume HIIT and higher-volume HIIT may produce similar improvements in VO_2_max (30), suggesting that for this outcome, greater total training volume does not necessarily translate into a linear increase in adaptive benefit. From the perspective of the present topic, which emphasizes the role of physical activity in diabetes and its complications, this finding further supports the practical value of HIIT as a time-efficient exercise strategy in adults with prediabetes.

#### 4.2.2 BMI

Most participants included in the present review were overweight or obese, and a higher BMI is widely recognized as being closely associated with prediabetes and its risk of progression (31). In the primary analysis, no statistically significant difference was observed between HIIT and MICT for reducing BMI. Although sensitivity analysis showed that heterogeneity decreased markedly after exclusion of an individual study, the direction and statistical significance of the pooled effect remained unchanged, indicating that the current evidence is insufficient to support a superiority of HIIT over MICT for improving BMI.

This finding may be partly explained by the short intervention duration of the included studies. Most trials lasted no longer than 2 weeks, and such short training periods are generally insufficient to induce substantial changes in body weight or BMI. In addition, BMI is a relatively crude anthropometric indicator and is insensitive to fat distribution, changes in lean mass, and body composition remodeling. As a result, it may underestimate the true effects of exercise interventions on body shape and metabolic phenotype.

Even so, the present finding still has some clinical relevance. In the absence of clear inferiority to MICT, HIIT achieved broadly comparable weight-related outcomes with less training time, suggesting potential feasibility and applicability in time-constrained populations. Previous studies have proposed that HIIT may influence anthropometric and body composition-related outcomes through mechanisms such as increased post-exercise energy expenditure, enhanced fat oxidation, and altered appetite regulation (32, 33). However, these effects are also likely to be influenced by dietary control, baseline adiposity, and intervention adherence. Future studies should therefore incorporate more sensitive indicators, such as fat mass, visceral adipose area, and lean mass, to better evaluate the effects of different exercise modalities on body shape and body composition.

#### 4.2.3 Blood pressure

Elevated blood pressure is common in adults with prediabetes and represents an important risk factor for atherosclerotic cardiovascular disease, heart failure, and microvascular complications (34). In the present meta-analysis, HIIT did not show a statistically significant advantage over MICT for improving either SBP or DBP. It should be noted, however, that heterogeneity was substantial for both outcomes, particularly for SBP, which was sensitive to individual studies, indicating that the current evidence has limited stability. Several factors may help explain these findings.

First, the number of included studies was limited, and most were small-sample trials, reducing the statistical power to detect between-group differences. Second, baseline blood pressure levels varied considerably across studies, and the magnitude of blood pressure reduction is often closely related to pre-intervention values; when baseline blood pressure is not elevated, between-group differences may be more difficult to detect. Third, differences in training parameters, intervention duration, and control of co-interventions, especially diet and habitual physical activity, may also have weakened the comparability of blood pressure effects across studies. Although evidence from other populations, such as adults with hypertension or prehypertension, has suggested that HIIT may confer greater benefit for some blood pressure outcomes (35, 36), these findings may not be directly generalizable to adults with prediabetes.

Taken together, the current evidence is better interpreted as indicating that HIIT and MICT have not yet shown a clear difference in blood pressure improvement in adults with prediabetes, and that further high-quality studies are needed to clarify this question.

#### 4.2.4 Blood lipids

Because only a limited number of studies reported lipid-related outcomes and the specific lipid indicators were not consistent across studies, no meta-analysis was performed for blood lipid outcomes and only a narrative synthesis was conducted. Overall, the available studies suggest that both HIIT and MICT may exert favorable effects on selected lipid parameters, which is broadly consistent with previous evidence showing that aerobic exercise can improve the lipid profile in general (37, 38). However, in adults with prediabetes, the current evidence remains insufficient to support a clear advantage of HIIT over MICT for improving blood lipids.

This interpretation is consistent with the findings presented in the Results section. Although some individual studies suggested that certain lipid parameters, such as TG or LDL-C, decreased more under one exercise modality than the other, the findings were inconsistent across studies and were not supported by a sufficient number of repeated studies with harmonized outcome reporting. In addition to training intensity and interval structure, lipid outcomes are likely to be influenced by dietary control, weight change, baseline metabolic status, and intervention adherence (32). Moreover, most studies reporting lipid outcomes were short-term interventions, which further limits the ability to judge stable changes in TC, TG, HDL-C, and LDL-C. Accordingly, the more appropriate interpretation at present is that both HIIT and MICT may improve selected lipid-related risk markers in adults with prediabetes, but there is still no stable evidence indicating which exercise modality is superior. Future studies should further standardize lipid outcome selection, reporting methods, and control of co-interventions in order to improve the comparability and interpretability of the evidence.

### 4.3 Strengths and limitations

This study systematically reviewed and meta-analyzed intervention studies investigating the effects of HIIT in adults with prediabetes, with a particular focus on glycemic control outcomes with clear clinical threshold relevance and cardiometabolic risk-related outcomes. Several strengths should be highlighted.

First, this review specifically focused on adults with prediabetes, a key intervention window, thereby avoiding the clinical heterogeneity introduced by combining prediabetes and type 2 diabetes populations in a single analysis. This population-specific approach may facilitate a more accurate understanding of the potential role of HIIT during the early stage of metabolic dysfunction. Second, in addition to the major glycemic outcomes, including FBG, 2hPG, and HbA1c, this review also incorporated cardiometabolic risk-related outcomes such as BMI, VO_2_peak/VO_2_max, blood pressure, and blood lipids, allowing a more comprehensive evaluation of the overall metabolic health effects of HIIT in adults with prediabetes. Third, in terms of data synthesis, the present review preferentially pooled change-from-baseline values, which may help reduce, at least to some extent, the influence of between-study baseline differences on effect estimation. In addition, this review adopted a relatively rigorous methodological framework, including prospective registration, PRISMA 2020 reporting, RoB 2 risk-of-bias assessment, and GRADE certainty evaluation, thereby enhancing the transparency and credibility of the findings. Finally, the present results suggest that the effects of HIIT are not uniform across outcomes, but instead appear to be outcome-specific, with potentially favorable signals particularly for cardiorespiratory fitness and selected post-challenge glycemic outcomes. This provides preliminary evidence to inform time-efficient exercise prescription and more targeted exercise recommendations in clinical practice.

Several limitations should also be acknowledged. First, only English-language studies were included, which may have introduced language bias and resulted in the omission of potentially relevant high-quality non-English studies. Second, although all included studies were randomized controlled trials, many provided insufficient reporting of sequence generation, allocation concealment, and analysis plans. In addition, strict blinding is difficult to implement in exercise intervention studies, which may increase the risk of bias and affect internal validity. Third, the overall sample sizes were small, and most interventions were short-term, with many lasting no longer than 2 weeks. This may have limited the ability to detect stable changes in outcomes such as HbA1c, BMI, blood pressure, and blood lipids, all of which often require longer intervention duration to change meaningfully.

Fourth, there was substantial heterogeneity in intervention characteristics across studies. Differences in exercise intensity definitions, interval structure, training volume, frequency, duration, and comparator conditions increased the complexity of pooled analysis and interpretation, and likely contributed to the high heterogeneity observed for several outcomes. Fifth, some studies did not adequately report or control for pre-intervention and during-intervention differences in participant characteristics, medication use, dietary management, and habitual physical activity, all of which may have influenced the comparison and interpretation of intervention effects. Sixth, the evidence base for several key outcomes remained limited. Outcomes such as HbA1c, blood pressure, and blood lipids were reported in relatively few studies, direct comparisons between HIIT and non-exercise controls were limited, and head-to-head comparisons among different HIIT protocols were also insufficient, thereby restricting the robustness and generalizability of the conclusions.

Seventh, several outcomes were sensitive to individual studies. Sensitivity analyses indicated that pooled results for outcomes such as FBG, 2hPG, SBP, and BMI were influenced to some extent by single studies, suggesting limited stability of the current evidence base. Eighth, blood lipids and some supplementary cardiometabolic outcomes could not be quantitatively synthesized because of the limited number of studies and inconsistent reporting, and could only be narratively summarized; therefore, the strength of evidence for these outcomes remains relatively weak. In addition, several included studies originated from closely related research groups and showed substantial similarity in participant characteristics, intervention duration, and protocol structure. Although the present review made every effort to treat them as independent studies where appropriate, caution is still warranted when interpreting overall sample characteristics and extrapolating the findings.

Accordingly, the findings of the present review are better understood as a stage-specific synthesis of the available evidence on HIIT in adults with prediabetes, rather than as a definitive ranking of the superiority of different exercise modalities.

## 5 Conclusion and implications

This systematic review and meta-analysis suggests that, in adults with prediabetes, the effects of HIIT may be outcome-specific. Compared with MICT, HIIT showed potentially favorable signals for improving 2hPG and cardiorespiratory fitness (VO_2_peak/VO_2_max), whereas MICT may be more advantageous for lowering FBG. For outcomes such as HbA1c, BMI, blood pressure, and blood lipids, no clear superiority of HIIT over MICT was observed based on the current evidence.

Overall, HIIT, as a time-efficient exercise strategy, appears to have potential practical value during this critical intervention window in prediabetes. However, the certainty of evidence for glycemic outcomes remains generally low, and the currently available evidence is insufficient to support definitive clinical judgments regarding the superiority of one exercise modality over another. Based on the present evidence, exercise prescription for adults with prediabetes should not rely on a simplistic recommendation of a single exercise modality, but should instead consider individual glycemic phenotype, priority outcome targets, and real-world feasibility. For individuals whose primary goals are improving post-challenge glycemic control and cardiorespiratory fitness, particularly when time is limited, HIIT may be a reasonable option to consider. In contrast, for those with greater emphasis on fasting glucose control, MICT may still remain an important choice.

Future research should be strengthened in several respects. First, more high-quality randomized controlled trials specifically targeting adults with prediabetes are needed, using consistent or at least comparable diagnostic criteria, intervention definitions, and outcome measures in order to improve the completeness and comparability of the evidence base. Second, study design and statistical analysis should more adequately address potential confounding factors, particularly baseline glycemic phenotype, medication use, dietary management, and habitual physical activity, in order to enhance internal validity and interpretability. Third, more direct comparisons are needed not only between HIIT and non-exercise controls or MICT, but also among different HIIT protocols, in order to clarify the differential effects of exercise modality and interval structure. Finally, future trials should consider longer intervention duration and improve the standardized reporting of HbA1c, blood pressure, blood lipids, and more sensitive anthropometric and body composition-related outcomes, so as to better evaluate the clinical applicability and translational potential of HIIT in adults with prediabetes.

Taken together, HIIT may be regarded as a time-efficient exercise strategy worthy of attention in adults with prediabetes, but its relative advantages over MICT still require confirmation from more high-quality evidence.

## Data Availability

The original contributions presented in this study are included in the article and/or **Supplementary Material**. The datasets analyzed in this study were derived from the full-text articles included in this systematic review and meta-analysis. Further inquiries can be directed to the corresponding author.
