# Supplementary material for: Effects of high-intensity interval training on glycemic control and cardiometabolic risk factors in adults with prediabetes: a systematic review and meta-analysis
Source: Front Endocrinol (Lausanne). 2026 May 14;17:1837386. doi: 10.3389/fendo.2026.1837386 (PMC13215823; doi:10.3389/fendo.2026.1837386)
Supplement: Supplementary file 1 [file Table1.docx]

Supplementary Table S1.

Search Strategy

The literature search was conducted independently in **PubMed, Cochrane Library, Embase, Web of Science, and EBSCO** from database inception to **January 2026**, and was limited to **English-language publications**. The search strategy combined terms related to **prediabetes**, **high-intensity interval training**, and **randomized controlled trials,** using both controlled vocabulary (e.g., MeSH, Emtree, where applicable) and free-text terms. In addition, the reference lists of included studies were manually screened to identify potentially eligible articles.

| **1. PubMed** |
| --- |
| (("Prediabetic State"[Mesh] OR prediabetes[Title/Abstract] OR impaired fasting glucose[Title/Abstract] OR impaired glucose tolerance[Title/Abstract] OR impaired glucose regulation[Title/Abstract] OR hyperglycemia[Title/Abstract] OR IFG[Title/Abstract] OR IGT[Title/Abstract] OR IGR[Title/Abstract]) AND ("High-Intensity Interval Training"[Mesh] OR high-intensity interval training[Title/Abstract] OR HIIT[Title/Abstract] OR HIIE[Title/Abstract] OR sprint interval training[Title/Abstract] OR SIT[Title/Abstract] OR REHIT[Title/Abstract] OR LVHIIT[Title/Abstract] OR HVHIIT[Title/Abstract]) AND (randomized controlled trial[Publication Type] OR randomized[Title/Abstract] OR randomised[Title/Abstract])) |
| **2. Cochrane Library** |
| ((MeSH descriptor: [Prediabetic State] explode all trees OR prediabetes:ti,ab,kw OR impaired fasting glucose:ti,ab,kw OR impaired glucose tolerance:ti,ab,kw OR IFG:ti,ab,kw OR IGT:ti,ab,kw) AND (MeSH descriptor: [High-Intensity Interval Training] explode all trees OR high-intensity interval training:ti,ab,kw OR HIIT:ti,ab,kw OR sprint interval training:ti,ab,kw  OR SIT:ti,ab,kw OR REHIT:ti,ab,kw) AND (randomized controlled trial:ti,ab,kw OR randomized:ti,ab,kw OR randomised:ti,ab,kw)) |
| **3. Embase** |
| (('prediabetes'/exp OR 'impaired fasting glucose'/exp OR 'impaired glucose tolerance'/exp OR prediabetes:ab,ti OR IFG:ab,ti OR IGT:ab,ti) AND ('high intensity interval training'/exp OR high-intensity interval training:ab,ti OR HIIT:ab,ti OR sprint interval training:ab,ti OR SIT:ab,ti OR REHIT:ab,ti) AND ('randomized controlled trial'/exp OR randomized:ab,ti OR randomised:ab,ti)) |
| **4. Web of Science** |
| TS = ((prediabetes OR impaired fasting glucose OR impaired glucose tolerance OR IFG OR IGT)  AND (high-intensity interval training OR HIIT OR sprint interval training OR SIT OR REHIT)  AND (randomized OR randomised OR "controlled trial")) |
| **5. EBSCO** |
| ((prediabetes OR impaired fasting glucose OR impaired glucose tolerance OR IFG OR IGT) AND  (high-intensity interval training OR HIIT OR sprint interval training OR SIT OR REHIT) AND  (randomized controlled trial OR randomized OR randomised)) |
