# Supplementary material for: Effects of high-intensity interval training on glycemic control and cardiometabolic risk factors in adults with prediabetes: a systematic review and meta-analysis
Source: Front Endocrinol (Lausanne). 2026 May 14;17:1837386. doi: 10.3389/fendo.2026.1837386 (PMC13215823; doi:10.3389/fendo.2026.1837386)
Supplement: Supplementary file 2 [file Table2.docx]

Supplementary Table S2.

Table 2 Inclusion criteria and extractable outcomes of the included studies

| Author | Year | Key inclusion criteria | Extractable outcomes in the present review |
| --- | --- | --- | --- |
| Robinson et al. | 2015 | HbA1c（ADA）➀➁➃ | FBG；BMI，VO₂peak，SBP/DBP |
| Jung et al. | 2015 | FBG/HbA1c（ADA）➀➂ | BMI，VO₂peak，SBP/DBP |
| Gilbertson et al. | 2018 | OGTT、HbA1c（ADA） | 2hPG；BMI，VO₂peak |
| Safarimosavi et al. | 2018 | FBG/OGTT（original study criteria） | FBG，2hPG，HbA1c |
| Gaitán et al. | 2019 | OGTT（ADA） | FBG，2hPG；BMI，VO₂peak |
| Eichner et al. | 2019 | OGTT（ADA） | BMI，VO₂peak，SBP/DBP |
| Malin et al. | 2020 | FBG、OGTT（ADA） | FBG，2hPG；BMI，VO₂peak |
| Heiston et al. | 2020 | FBG、OGTT（ADA）➁ | FBG，2hPG；BMI，VO₂peak，SBP/DBP，TG，HDL-C，LDL-C |
| Badaam et al. | 2021 | FBG、OGTT（ADA） | FBG，HbA1c；BMI |
| Eichner et al. | 2021 | OGTT（ADA） | FBG，2hPG；BMI，VO₂peak |
| Battillo et al. | 2023 | FBG、OGTT、HbA1c（ADA） | BMI，VO₂peak，SBP/DBP，lipid profile |
| Malin et al. | 2023 | FBG、OGTT（ADA） | FBG，2hPG；BMI，VO₂peak |
| Chen et al. | 2025 | FBG、2hPG、HbA1c（original study criteria） | FBG，2hPG，HbA1c；BMI，VO₂peak，SBP/DBP，TC，TG，HDL-C，LDL-C |

Note: ➀ diabetes risk questionnaire assessment； ➁ BMI-based eligibility criterion；➂ physician diagnosis；➃ sedentary lifestyle。ADA = American Diabetes Association；OGTT = oral glucose tolerance test；FBG = fasting blood glucose；FPG = fasting plasma glucose；2hPG = 2-hour postprandial glucose；HbA1c = glycated hemoglobin；BMI = body mass index；VO₂peak = peak oxygen uptake；VO₂max = maximal oxygen uptake；SBP = systolic blood pressure；DBP = diastolic blood pressure；TC = total cholesterol；TG = triglycerides；HDL-C = high-density lipoprotein cholesterol；LDL-C = low-density lipoprotein cholesterol。
