# Supplementary material for: Effects of high-intensity interval training on glycemic control and cardiometabolic risk factors in adults with prediabetes: a systematic review and meta-analysis
Source: Front Endocrinol (Lausanne). 2026 May 14;17:1837386. doi: 10.3389/fendo.2026.1837386 (PMC13215823; doi:10.3389/fendo.2026.1837386)
Supplement: Supplementary file 3 [file Table3.docx]

Supplementary Table S3.

Table S3. Characteristics of the exercise programs in the included studies

| Author | Year | Group | Frequency / Duration | Session duration | Exercise prescription |
| --- | --- | --- | --- | --- | --- |
| Robinson et al. | 2015 | HIIT | 10 sessions / 2 weeks | 14–26 min | 3-min warm-up; 4–10 × (1 min at 85%–90% HR_peak + 1 min recovery); 3-min cool-down |
|  |  | MICT | 10 sessions / 2 weeks | 20–50 min | 3-min warm-up; continuous exercise at 60%–65% HR_peak; 3-min cool-down |
| Jung et al. | 2015 | HIIT | 12 sessions / 4 weeks | 25 min | 3-min warm-up; 10 × (1 min at 90% HR_peak + 1 min recovery); 2-min cool-down |
|  |  | MICT | 12 sessions / 4 weeks | 50 min | 3-min warm-up; 50 min continuous exercise at approximately 65% HR_peak; 2-min cool-down |
| Gilbertson et al. | 2018 | HIIT | 12 sessions / 13 days | 60 min | 10 × (3 min at 90% HR_peak + 3 min at 50% HR_peak) |
|  |  | MICT | 12 sessions / 13 days | 60 min | 60 min continuous exercise at 70% HR_peak |
| Safarimosavi et al. | 2018 | HIIT | 48 sessions / 12 weeks | 30 min | 5-min warm-up; 10 × (1 min at 90% VO₂peak + 1 min recovery); 5-min cool-down |
|  |  | CON | — | — | Non-exercise control |
| Gaitán et al. | 2019 | HIIT | 12 sessions / 13 days | 60 min | 10 × (3 min at 90% HR_peak + 3 min at 50% HR_peak) |
|  |  | MICT | 12 sessions / 13 days | 60 min | 60 min continuous exercise at 70% HR_peak |
| Eichner et al. | 2019 | HIIT | 12 sessions / 13 days | 60 min | 10 × (3 min at 90% HR_peak + 3 min at 50% HR_peak) |
|  |  | MICT | 12 sessions / 13 days | 60 min | 60 min continuous exercise at 70% HR_peak |
| Malin et al. | 2020 | HIIT | 12 sessions / 13 days | 60 min | 10 × (3 min at 90% HR_peak + 3 min at 50% HR_peak) |
|  |  | MICT | 12 sessions / 13 days | 60 min | 60 min continuous exercise at 70% HR_peak |
| Heiston et al. | 2020 | HIIT | 12 sessions / 13 days | 60 min | 10 × (3 min at 90% HR_peak + 3 min at 50% HR_peak) |
|  |  | MICT | 12 sessions / 13 days | 60 min | 60 min continuous exercise at 70% HR_peak |
| Badaam et al. | 2021 | HIIT | 36 sessions / 12 weeks | 30 min | 15-min warm-up; 4 × (1 min all-out sprint + 90 s recovery); 5-min cool-down |
|  |  | MICT | 60 sessions / 12 weeks | 40 min | 5-min warm-up; 30 min brisk walking; 5-min cool-down |
| Eichner et al. | 2021 | HIIT | 12 sessions / 13 days | 60 min | 10 × (3 min at 90% HR_peak + 3 min at 50% HR_peak) |
|  |  | MICT | 12 sessions / 13 days | 60 min | 60 min continuous exercise at 70% HR_peak |
| Battillo et al. | 2023 | HIIT | 12 sessions / 13 days | 60 min | 10 × (3 min at 90% HR_peak + 3 min at 50% HR_peak) |
|  |  | MICT | 12 sessions / 13 days | 60 min | 60 min continuous exercise at 70% HR_peak |
| Malin et al. | 2023 | HIIT | 12 sessions / 13 days | 60 min | 10 × (3 min at 90% HR_peak + 3 min at 50% HR_peak) |
|  |  | MICT | 12 sessions / 13 days | 60 min | 60 min continuous exercise at 70% HR_peak |
| Chen et al. | 2025 | HIIT | 36 sessions / 12 weeks | 30 min | 5-min warm-up; 10 × (1 min at 75%–90% HR_peak + 1 min recovery); 5-min cool-down |
|  |  | MICT | 36 sessions / 12 weeks | 60 min | 5-min warm-up; 50 min continuous exercise at 55%–70% HR_peak; 5-min cool-down |

Note: Except for Robinson et al. and Jung et al., which used self-selected aerobic exercise modalities, and the MICT group in Badaam et al., which involved brisk walking, most studies used cycle ergometer training. HIIT, high-intensity interval training; MICT, moderate-intensity continuous training; CON, control group; HR_peak, peak heart rate; VO₂peak, peak oxygen uptake.
