# Supplementary material for: Effects of high-intensity interval training on glycemic control and cardiometabolic risk factors in adults with prediabetes: a systematic review and meta-analysis
Source: Front Endocrinol (Lausanne). 2026 May 14;17:1837386. doi: 10.3389/fendo.2026.1837386 (PMC13215823; doi:10.3389/fendo.2026.1837386)
Supplement: Supplementary file 4 [file Table4.docx]

Supplementary Table S4.

Contextual control and background factors of included studies

| **Study** | **Dietary control** | **Medication use** | **Baseline physical activity** | **Other relevant background factors** | **Remarks** |
| --- | --- | --- | --- | --- | --- |
| Robinson et al., 2015 | No strict dietary control reported; standardized fasting/testing procedures were described | Baseline medication information reported; glucose-lowering medications excluded | Participants were inactive/low active at baseline | Supervised exercise intervention; testing procedures standardized | Background control partially reported rather than fully standardized. |
| Jung et al., 2015 | No strict dietary control clearly reported | Not clearly reported | Participants were low active/inactive at baseline | Home-based training with behavioral support, logs, and heart-rate monitoring | Greater emphasis on adherence/behavioral support than metabolic standardization. |
| Safarimosavi et al., 2018 | Participants were instructed to maintain habitual diet; 3-day food records were collected and replicated before post-testing; alcohol/caffeine restriction reported | Participants were medication-free | Habitual lifestyle maintained; no physical activity 24 h before testing | Isocaloric protocols; HR monitored; repeated exercise testing used to adjust prescriptions | Dietary intake was monitored, but not tightly controlled by investigators. |
| Gilbertson et al., 2018 | Mixed diet maintained; participants recorded diet; approximately 250 g carbohydrate before OGTT; caffeine/alcohol/medication restrictions before testing | Medications affecting insulin sensitivity excluded | Sedentary; excluded if >60 min/week structured exercise | Phenotype-stratified/block randomization; fully supervised, work-matched training | Good control of pre-test metabolic conditions. |
| Gaitán et al., 2019 | Participants were instructed not to change diet; food logs collected; approximately 250 g carbohydrate before OGTT; restrictions before testing | Medications affecting substrate metabolism, blood flow, or insulin sensitivity excluded | Sedentary; <60 min/week structured exercise | Fully supervised isocaloric intervention; post-testing conducted ~24 h after last session | Good pre-test standardization despite ad libitum diet during the intervention. |
| Eichner et al., 2020 | Approx. 250 g carbohydrate before testing; 3-day food logs; alcohol/medication/supplement restrictions before testing | Medications affecting insulin sensitivity or vascular function excluded | Excluded if physically active >60 min/week | 12 supervised, work-matched sessions; post-test ~24 h after last bout | Dietary sugar intake was additionally analyzed and discussed as a modifier. |
| Malin et al., 2019 | Approx. 250 g carbohydrate before testing; diet recorded and replicated; 3-day food logs; alcohol/caffeine/medication restrictions before visits | Medications affecting endothelial function or insulin sensitivity excluded | Excluded if exercise >60 min/week | Work-matched supervised training; post-test ~24 h after last session | Contextual control was moderate to good, especially for pre-test standardization. |
| Heiston et al., 2020 | Approx. 250 g carbohydrate before testing; habitual diet maintained during intervention; 3-day food logs collected | Anti-diabetic or weight-inducing medications excluded | Physically inactive (≤60 min/week) | Block-randomized supervised training; HR and RPE monitored | Good reporting of metabolic and behavioral context. |
| Badaam et al., 2021 | Participants were asked to maintain usual dietary habits | Prediabetes pharmacotherapy excluded; some attrition due to starting metformin | Regular exercisers/sports participants excluded | Field-based real-life intervention; compliance threshold >80%; post-test 48–72 h after last session | Contextual control was pragmatic rather than laboratory-standardized. |
| Battillo et al., 2023 | Approx. 250 g carbohydrate before testing; diet recorded and replicated; habitual diet and non-exercise activity maintained | Anti-diabetic or weight-inducing medications excluded | Sedentary; <60 min/week | Block-randomized, supervised, work-matched training | Good control of pre-test conditions and exercise exposure. |
| Malin and Syeda, 2024 | Dietary intake recorded; ~250 g carbohydrate before testing; alcohol/caffeine/activity/medication restrictions before visits | Medications affecting weight, insulin sensitivity, or vascular function excluded | Sedentary; ≤60 min/week structured exercise | 12 supervised, work-matched sessions over 13 days | Strong metabolic control around testing sessions. |
| Chen et al., 2025 | All participants received dietary recommendations based on the Dietary Guidelines for Chinese Residents (2022) to control total energy intake and dietary balance | Not clearly reported in the available text | No regular exercise for at least 3 months before enrollment | Assessor-blind RCT; adherence monitored; HR recorded during training | More structured dietary advice was provided than in most other studies. |
| Eichner et al., 2019 | Diet recorded and replicated; ~250 g/day carbohydrate before testing; caffeine/alcohol/medication/supplement restrictions | Medications influencing endothelial function or insulin sensitivity excluded; limited background medication use reported | Physically inactive; <60 min/week exercise | Supervised, work-matched training; exercise dose estimated as kcal/session | Good reporting of pre-test diet standardization and exercise dose. |

Abbreviations: HR, heart rate; OGTT, oral glucose tolerance test; RPE, rating of perceived exertion.

Note: This table summarizes contextual control and background factors that may influence the interpretation of glycemic and cardiometabolic outcomes, including dietary control, medication use, and baseline physical activity. “Not clearly reported” indicates that the relevant information was not explicitly described in the available full text.
