# Supplementary figures and images for: Effects of high-intensity interval training on glycemic control and cardiometabolic risk factors in adults with prediabetes: a systematic review and meta-analysis
Source: Front Endocrinol (Lausanne). 2026 May 14;17:1837386. doi: 10.3389/fendo.2026.1837386 (PMC13215823; doi:10.3389/fendo.2026.1837386)

| Supplementary Table S5. |
| --- |


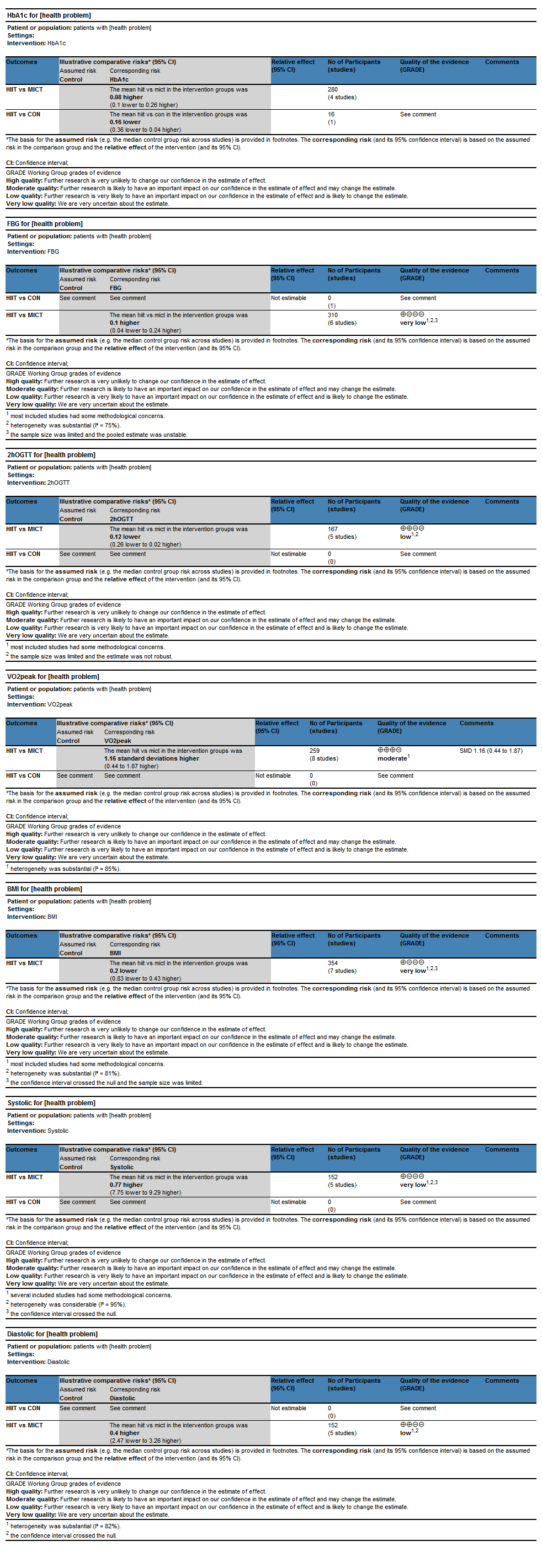

Supplement: Supplementary file 5 [file Table5.docx]
